# Wake Forest University long‐term follow‐up of type 2 myocardial infarction: The Wake‐Up T2MI Registry

**DOI:** 10.1002/clc.23182

**Published:** 2019-04-12

**Authors:** Hanumantha R. Jogu, Sameer Arora, Muthiah Vaduganathan, Arman Qamar, Ambarish Pandey, Parag A. Chevli, Tusharkumar H. Pansuriya, Muhammad I. Ahmad, Abhishek Dutta, Padageshwar R. Sunkara, Waqas Qureshi, Sujethra Vasu, Bharathi Upadhya, Deepak L. Bhatt, James L. Januzzi, David Herrington

**Affiliations:** ^1^ Department of Internal Medicine Wake Forest School of Medicine Winston‐Salem North Carolina; ^2^ Division of Cardiology University of North Carolina Chapel Hill North Carolina; ^3^ Brigham and Women's Hospital Heart & Vascular Center Harvard Medical School Boston Massachusetts; ^4^ Department of Cardiology University of Texas Southwestern Medical Center Dallas Texas; ^5^ Division of Cardiovascular Medicine, Department of Internal Medicine University of Massachusetts School of Medicine Worcester Massachusetts; ^6^ Section on Cardiovascular Medicine Wake Forest School of Medicine Winston‐Salem North Carolina; ^7^ Cardiology Division, Massachusetts General Hospital, and Cardiometabolic Trials Baim Institute for Clinical Research Boston Massachusetts

**Keywords:** acute myocardial injury, chronic myocardial injury, demand ischemia, myonecrosis, troponin, type 2 myocardial infarction

## Abstract

**Background:**

The Wake‐Up T2MI Registry is a retrospective cohort study investigating patients with type 2 myocardial infarction (T2MI), acute myocardial injury, and chronic myocardial injury. We aim to explore risk stratification strategies and investigate clinical characteristics, management, and short‐ and long‐term outcomes in this high‐risk, understudied population.

**Methods:**

From 1 January 2009 to 31 December 2010, 2846 patients were identified with T2MI or myocardial injury defined as elevated cardiac troponin I with at least one value above the 99th percentile upper reference limit and coefficient of variation of 10% (>40 ng/L) and meeting our inclusion criteria. Data of at least two serial troponin values will be collected from the electronic health records to differentiate between acute and chronic myocardial injury. The Fourth Universal Definition will be used to classify patients as having (a) T2MI, (b) acute myocardial injury, or (c) chronic myocardial injury during the index hospitalization. Long‐term mortality data will be collected through data linkage with the National Death Index and North Carolina State Vital Statistics.

**Results:**

We have collected data for a total of 2205 patients as of November 2018. The mean age of the population was 65.6 ± 16.9 years, 48% were men, and 64% were white. Common comorbidities included hypertension (71%), hyperlipidemia (35%), and diabetes mellitus (30%). At presentation, 40% were on aspirin, 38% on β‐blockers, and 30% on statins.

**Conclusion:**

Improved characterization and profiling of this cohort may further efforts to identify evidence‐based strategies to improve cardiovascular outcomes among patients with T2MI and myocardial injury.

**ABBREVIATIONS**
CAD: coronary artery disease
cTnI: cardiac troponin I
CTSI: Clinical and Translational Science Institute
CV: cardiovascular
ICD: International Classification of Disease
REDCap: Research Electronic Data Capture
T1MI: type 1 myocardial infarction
T2MI: type 2 myocardial infarction
WFUBMC: Wake Forest University Baptist Medical Center

## 1 INTRODUCTION

Myocardial necrosis due to myocardial ischemia is designated as myocardial infarction (MI). Recognizing the heterogeneity inherent to this entity, in 2007 and 2012, the ESC/ACCF/AHA/WHF Task Force for the Universal Definition of MI released expert consensus documents redefining MI into five types.^1^ This classification system was recently further refined with the Fourth Universal Definition of MI.^2^ Type 1 myocardial infarction (T1MI) refers to acute coronary syndrome (ACS) caused by atherosclerotic plaque rupture, ulceration, fissure, or erosion leading to intraluminal thrombus formation and obstructed coronary blood flow. Type 2 myocardial infarction (T2MI) was defined as myocardial ischemia, not due to plaque rupture but secondary to an imbalance between myocardial oxygen demand and/or supply due to an underlying cause.^2^ In defining presence of MI, a critical characteristic is presence of myocardial ischemia; this may be manifested by symptoms, changes on electrocardiography, or evidence for loss of myocardial function. Patients with evidence of elevated troponin with at least one value above the 99th percentile upper reference limit (URL) and 10% coefficient of variation without overt myocardial ischemia are classified as having myocardial injury.^2^ This injury may be acute or chronic, depending on the pattern of cTn elevation and in appropriate clinical contexts.

T2MI and myocardial injury are commonly encountered in clinical practice. In smaller studies, T2MI has been found to be responsible for 2% to 37% of all elevated troponin results in unselected hospitalized patients and 5% to 71% in an unselected emergency department setting.^3^, ^4^, ^5^, ^6^, ^7^ Similarly, myocardial injury has been reported in up to 70% of unselected patients.^8^, ^9^ The heterogeneity in reported frequencies across studies is likely due to differences in biomarkers cut‐offs, selected populations, variation in adjudication processes, and challenges in clinically distinguishing myocardial injury from infarction.

Short‐ and long‐term event rates are higher among patients who experience T2MI compared with patients with T1MI^1^, ^3^, ^4^, ^8^, ^10^, ^11^, ^12^, ^13^, ^14^, ^15^, ^16^, ^17^, ^18^, ^19^, ^20^, ^21^, ^22^, ^23^, ^24^, ^25^, ^26^, ^27^, ^28^ (Table 1). Although the role of underlying coronary artery disease (CAD) and microvascular disease remains unclear, it may play an essential role in influencing outcomes in T2MI.^10^, ^29^ Rates of obstructive CAD in T2MI patients undergoing coronary angiography range from 28% to 78%.^3^, ^8^, ^9^, ^27^ Despite a high prevalence of CAD in these patients, significant disparities exist in medical management of even those with CAD in T2MI or myocardial injury, when compared with those with T1MI^1^, ^3^, ^4^, ^8^, ^9^, ^10^, ^11^, ^12^, ^14^, ^15^, ^19^, ^20^, ^21^, ^22^, ^23^, ^24^, ^25^, ^26^, ^27^ (Table 2).

**Table 1:** Mortality rate of T2MI and myocardial injury in selected studies

Study	N	Mortality of T1MI patients	Mortality of T2MI patients	Mortality of myocardial injury patients	Mortality of T2MI vs T1MI patients	Mortality of myocardial injury vs T1MI patients	Mortality of T2MI vs myocardial injury
Putot et al (2018)^19^	4436 (conventional cTnI ≥10 μg/L)	125 (6.1%) in‐hospital mortality	133 (14%) in‐hospital mortality	260 (17.9%) in‐hospital mortality			
Lambrecht et al (2018)^20^	1568 (cTnI >30 ng/L)	114 of 360 (32%) at 3.2 years. CVD: 68 (61.3%)	74 of 119 (62%) at 3.2 years. CVD: 29 (42.6%)	639 of 1089 (59%) at 3.2 years. CVD: 252 (41.2%)	NA	NA	No difference T2MI vs myocardial injury
Smilowitz et al (2018)^21^	710 (cTnI >0.06 ng/mL)	41 (29.9%) of 137 at 1.8 years. 18 (13%) in‐hospital death	45 (30.8%) of 146 at 1.8 years. 17 (12%) in‐hospital death	52 (29.7%) of 175 at 1.8 years. 16 (9%) in‐hospital			No difference T2MI vs myocardial injury
Arora et al (2018)^22^	1039 (NSTEMI)	12.4% of 775 at 1 year	34.9% of 264 at 1 year		2.80 (2.13‐3.67) RR (95% CI)		
Chapman et al (2018)^10^	2122 (cTnI ≥0.05 μg/L)	430 (37%) at 4.9 years all‐cause. CVD: 253 (22%)	268 (63%) at 4.9 years all‐cause. CVD: 104 (24%)	378 (72%) at 4.9 years all‐Cause. CVD: 145 (28%)	1.51 (1.21‐1.87) RR (95% CI). Adjusted age, sex, renal function, Hb, smoking, diabetes, HTN, CAD, stroke, PVD. 2.15 (1.82‐2.55) unadjusted	2.09 (1.72‐2.55) RR (95% CI). Adjusted age, sex, renal function, Hb, smoking, diabetes, HTN, CAD, stroke, PVD. 2.88 (2.43‐3.40) unadjusted	1.27 (1.08‐1.48) adjusted RR (95% CI). Excess all‐cause mortality of myocardial injury vs T2MI. No difference T2MI vs myocardial injury for CVD
Sandoval et al (2017)^23^	1640 (cTnI value sex specific >99th percentile)	6 (8%) all‐cause mortality at 180 days	18 (13%) all‐cause mortality at 180 days	30 (11%) all‐cause mortality at 180 days			No difference T2MI vs myocardial injury
Cediel et al (2017)^24^	1010 (cTnI >0.039 μg/mL corresponds to 99th percentile URL with coefficient of variation <10%)	74 (19.7%) at 2 years	77 (39.7%) at 2 years	176 (40%) at 2 years	1.41 (1.02‐1.94) HR (95% CI)	1.54 (1.16‐2.04) HR (95% CI)	No difference T2MI vs myocardial injury
Gaggin et al (2017)^26^	1251 (>99th percentile URL or lowest cTn with <10% imprecision)		23.3% per 100 person‐years	3.3% per 100 person‐years in non‐T2MI	2.96 (2.01‐4.36) HR (95% CI) T2MI vs without T2MI		
Sarkisian et al (2016)^11^	1577 (99th percentile URL and coefficient if variation of 10% was >30 ng/L of cTnI)	115 (31%) of 369 at 3.2 years	75 (63%) of 119 at 3.2 years	645 (59%) of 1089 at 3.2 years			1.28 (0.97‐1.65) HR (95% CI). No difference T2MI vs myocardial injury
Smilowitz et al (2016)^25^	475 (>99th percentile URL of cTn)		16 (6%) of all‐cause in‐hospital mortality	10 (5%) of all‐cause in‐hospital mortality			No difference T2MI vs myocardial injury
Shah et al (2015)^12^	2165 (cTnI ≥50 ng/L)	187 (16%) of 1171 at 1 year	134 (37%) of 429 at 1 year	193 (37%) of 522 at 1 year	1.95 (1.61‐2.37) RR (95% CI)	2.36 (1.990‐2.81) RR (95% CI)	1.19 (0.99‐1.42) RR (95% CI). No difference T2MI vs myocardial injury
Baron et al (2015)^13^	19 763 (AMI patients)	13.5% at 1 year	24.7% at 1 year		1.86 (1.66‐2.08) HR (95% CI) unadjusted at 1 year. Adjusted with age, sex, comorbidities, treatment and cTnI level 1.03 (0.86‐1.23)		
Spatz et al (2015)^27^	2082 (AMI patients)	53 (2.2%) at 1 year	3 (2.4%) at 1 year				
Sandoval et al (2014)^4^	1112 (cTnI >34 ng/L corresponds to 99th percentile URL and 10% coefficient of variation)	7.60% death at 180 days	11.4% deaths at 180 days				
Saaby et al (2013, 2014)^9^, ^15^	553 (cTnI >0.03 μg/L)	92 (25.6%) of 360 at 2.1 years	58 (48.7%) of 119 at 2.1 years		2.3 (1.7‐3.3) HR (95% CI) univariable Cox regression analyses		
Stein et al (2014)^14^	2818 (ACS patients)	8.6% at 1 year	23.9% at 1 year				
El‐Haddad (2012)^28^	807 (cTnI ≥1.6 ng/mL)	28 (5.4%) of 512 at 1 year	84 (28.5%) of 295 at 1 year				
Javed et al (2009)^8^	216 (cTnI >0.04 ng/mL)	15 (11%) of 143 in‐hospital mortality	9 (14%) of 64 in‐hospital mortality	67 (15%) of 461 non‐MI group in‐hospital mortality			

Abbreviations: ACS, acute coronary syndrome; AMI, acute myocardial infarction; CAD, coronary artery disease; CI, confident interval; cTnI, cardiac troponin I; cTnT, cardiac troponin T; CVD, cardiovascular death; Hb, hemoglobin; HR, hazard ratio; HTN, hypertension; MI, myocardial infarction; NA, not available; NSTEMI, non‐ST elevation myocardial infarction; PVD, peripheral vascular disease; RR, relative risk; T1MI, type 1 myocardial infarction; T2MI, type 2 myocardial infarction.

**Table 2:** Frequency and CAD prevalence of T2MI and myocardial injury in selected studies

Study	Classification of T2MI and myocardial injury patients	Population	N	Number (%) of T2MI	Number (%) of myocardial injury	Prevalence of CAG and obstructive CAD of T2MI patients	Prevalence of CAG and obstructive CAD of myocardial injury patients	Troponin assay
Putot et al (2018)^19^	Adjudicated by clinician in charge, secondary adjudicated by two cardiologists	Observational prospective study of patients who come to ER university hospital	4436 (conventional cTnI ≥10 μg/L)	947 (21.3%)	1453 (32.8%)	325 (34.3%) had CAG		Siemens Dimension Vista (Siemens Healthineers, Erlangen, Germany)
Arora et al (2018)^22^	Adjudicated by single user	All NSTEMI diagnosed patients of single center study	1039 (NSTEMI)	264 (25.4%) of 1039 NSTEMI patients	NA	68 out of 264 had CAG	NA	Siemens Healthcare Diagnostics
Smilowitz et al (2018)^21^	Classified by consulting and cardiologist and reviewing documentation	Retrospective single center study	710 (cTnI >0.06 ng/mL)	146 (21%)	175 (25%)	19 out of 146 had CAG of which 15 (78.9%) had significant obstruction	19 out of 175 had CAG of which 14 (73.7%) had significant obstruction	Troponin‐Ultra assay (Siemens Healthcare Diagnostics, Erlangen, Germany)
Lambrecht et al (2018)^20^	Adjudicated by three cardiologists	Prospective DEF‐AMI study of all hospitalized patients with elevated cTnI	1568 (cTnI >30 ng/L)	119 (3%) of 1568 patients	1089 (29%) of 1568 patients	28 (23.5%) out of 119 had CAG	57 (5.2%) out of 1089 had CAG	Architect C16000 (Abbott Diagnostics, Abbott Park, III)
Cediel et al (2017)^24^	Adjudicated by two cardiologists according to the Third Universal Definition	Retrospective cohort study in university hospital	1010 (cTnI >0.039 μg/mL corresponds to 99th percentile URL with coefficient of variation <10%)	194 (19.2%) of 1010	440 (43.6%) of 1010	11 out of 194 (5.67%) had CAG	23 out of 440 (5.23%) had CAG	cTnI‐Ultra from Siemens, Advia Centaur
Sandoval et al (2017)^23^	Adjudicated according to Third Universal Definition by two clinicians. Third senior in case of discrepancy	Consecutive unselected patients from emergency department	1640 (cTnI value sex‐specific >99th percentile)	140 (8.5%)	280 (17%)	13 (9%) out of 140 had CAG of which 7 (54%) had significant obstruction	8 (3%) out of 280 had CAG of which 0 had significant obstruction	Hs‐cTnI assays on Abbott ARCHITECT i1000sr or i2000sr analyzers (Abbott, Abbott Park, Ill)
Gaggin et al (2017)^26^	Group of investigators comprising cardiologist. Medical records review based on Third Universal Definition of MI	Single center prospective cohorts (CASABLANCA) study in consecutives patients undergoing coronary and peripheral angiographic procedure	1251 (>99th percentile URL or lowest cTn with <10% imprecision)	152 (12.2%)		61.2% had ≥50% coronary stenosis in ≥2 vessels		Roche Diagnostics Troponin T STAT assay and Roche Cobas e 601 analyzer
Smilowitz et al (2016)^25^	Retrospective review of medical record	Single center retrospective charge review	475 (>99th percentile URL of cTn)	255 (54%)	220 (46%)	20 (8%) had CAG of which 19 had CAD	5 (2%) had CAG of which 4 had CAD	VITROS cTnI ES assay (Ortho‐Clinical Diagnostics, Rochester, NY) or the ST AIA‐PACK 2ng generation (Tosoh Bioscience, Tokyo, Japan)
Sarkisian et al (2016)^11^	Classified by three cardiologists	Prospective study of unselected hospital patients who had a cTnI measured on clinical indication	1577 (cTnI >30 ng/L corresponds to 99th percentile URL and coefficient of variation of 10%)	119 (8%) of total 1577	1089 of (69%) of total 1577 patients with cTnI >0.03 μg/L	28 out of 119 had CAG of which 15 (54%) had significant obstruction	57 out of 1089 had CAG of which 30 (52.6%) had significant obstruction	cTnI Architect c16000. Abbott Diagnostics
Baron et al (2016)^3^	Classified by treating physicians. Included patients with infection	Consecutive patients with MI admitted to cardiac units at 73 hospitals over 3 years	59 394 (AMI patients)	4083 (6.9%)	1549 (2.6%) Unclassified MI	1316 out of 4083 (32.2%) had CAG, of which 52.8% had obstructive CAD	NA	Results are reported for both hs‐cTnT and cTnI, without details
Spatz et al (2015)^27^	Classified by team of five physicians	Prospective cohort study of variation in recovery: Role of gender on outcomes of young AMI patients (VIRGO)	2082 (AMI patients)	123 (5.9%)	NA	35 (28.5%) of 123 had significant obstruction	NA	NA
Shah et al (2015)^12^	Adjudicated diagnoses of T2MI. Adjudicated diagnoses of myocardial injury	Consecutive patients admitted with plasma cTnI values ≥50 ng/L	2165 (cTnI ≥50 ng/L)	429 (26.1%) of total 1643 MI or 19.8% of total 2165 patients	522 (24.1%) of total 2165 patients with cTnI ≥50 ng/L	31 out of 419 (7%) had CAG	19 out of 522 (4%) had CAG	ARCHITECT cTnI assay
Saaby et al (2013, 2014)^9^, ^15^	Adjudicated T2MI diagnoses based on oxygen supply/demand mismatch supplemented by specific clinical standards. cTnI >0.03 μg/L but without overt myocardial ischemia were classified as myocardial injury	Prospective study of unselected hospital patients who had a cTnI measured on clinical indication	553 (cTnI >0.03 μg/L)	144 (26%) of total of 553 MI patients	1408 (71.8%) of total 1961 patients with cTnI >0.03 μg/L	31 out of 144 (21.5%) had CAG of which 17 (54.8%) had significant CAD	56 out of 1089 (5.14%) had CAG of 33 (59.9%) had significant CAD	cTnI Architect c16000. Abbott Diagnostics
Sandoval et al (2014)^4^	Adjudicated by two clinicians based on Universal definition of MI consensus document	Retrospective unselected consecutive patients	1112 (cTnI >34 ng/L corresponds to 99th percentile URL and 10% coefficient of variation)	190 (17%) of 1112 total patients	856 (77%) of total 1112 patients	17 out of 190 had CAG	28 out of 856 had CAG	Ortho‐Clinical Diagnostics (OCD) VITROS cTnI ES assay
Stein et al (2014)^14^	Retrospective validation of T2MI by 2 physicians. Included patients with infection	Prospective collection of patients with ACS	2818 (ACS patients)	127 (4.5%)	NA	36.2% had CAG of which 50% underwent PCI	NA	Details on cTn assays are not reported
Javed et al (2009)^8^	Classified by the interviewing physicians	Prospective evaluation of large cohort	701 (cTnI >0.04 ng/mL)	64 (29.6%)	461 (65.8%)	32 out of 64 (50%) had CAG of which 25 (78%) had significant obstruction	150 out of 461 had CAG of which 78 (52%) had significant obstruction	ADVIA Immunoassay Systems cTnI Ultra assay, Siemens Healthcare Diagnostics

Abbreviations: ACS, acute coronary syndrome; AMI, acute myocardial infarction; CAD, coronary artery disease; CAG, coronary angiography; cTnI, cardiac troponin I; cTnT, cardiac troponin T; MI, myocardial infarction; NA, not available; NSTEMI, non‐ST elevation myocardial infarction; T1MI, type 1 myocardial infarction; T2MI, type 2 myocardial infarction.

Relatively few studies are available comprehensively characterizing the longitudinal profile, medical and interventional management, and short‐ and long‐term clinical prognosis of patients with T2MI or myocardial injury.^26^ There is a lack of consensus on the optimal therapeutic approach to this heterogeneous cohort of patients, including whether they benefit similarly from guideline‐based ACS therapies (as T1MI).^30^ Although select studies have characterized patients with T2MI, patients with myocardial injury have been infrequently studied. Few studies have leveraged linked national and state death records to facilitate more complete mortality estimates.

Our study has the following objectives: (a) to explore clinical characteristics of patients with T2MI and myocardial injury; (b) to investigate the differences in presentation, stratified by age, sex, and race; (c) to characterize utilization of noninvasive and invasive ischemic evaluation strategies in this population; (d) to determine the rates and burden of obstructive CAD; (e) to determine differences in medical and interventional management of T2MI and myocardial injury; (f) to investigate causes of cardiovascular and noncardiovascular mortality in T2MI and myocardial injury; (g) to identify predictors of in‐hospital, 180‐days, 1‐year, 5‐year, and 7‐year outcomes.

## 2 METHODS

**Figure 1:**
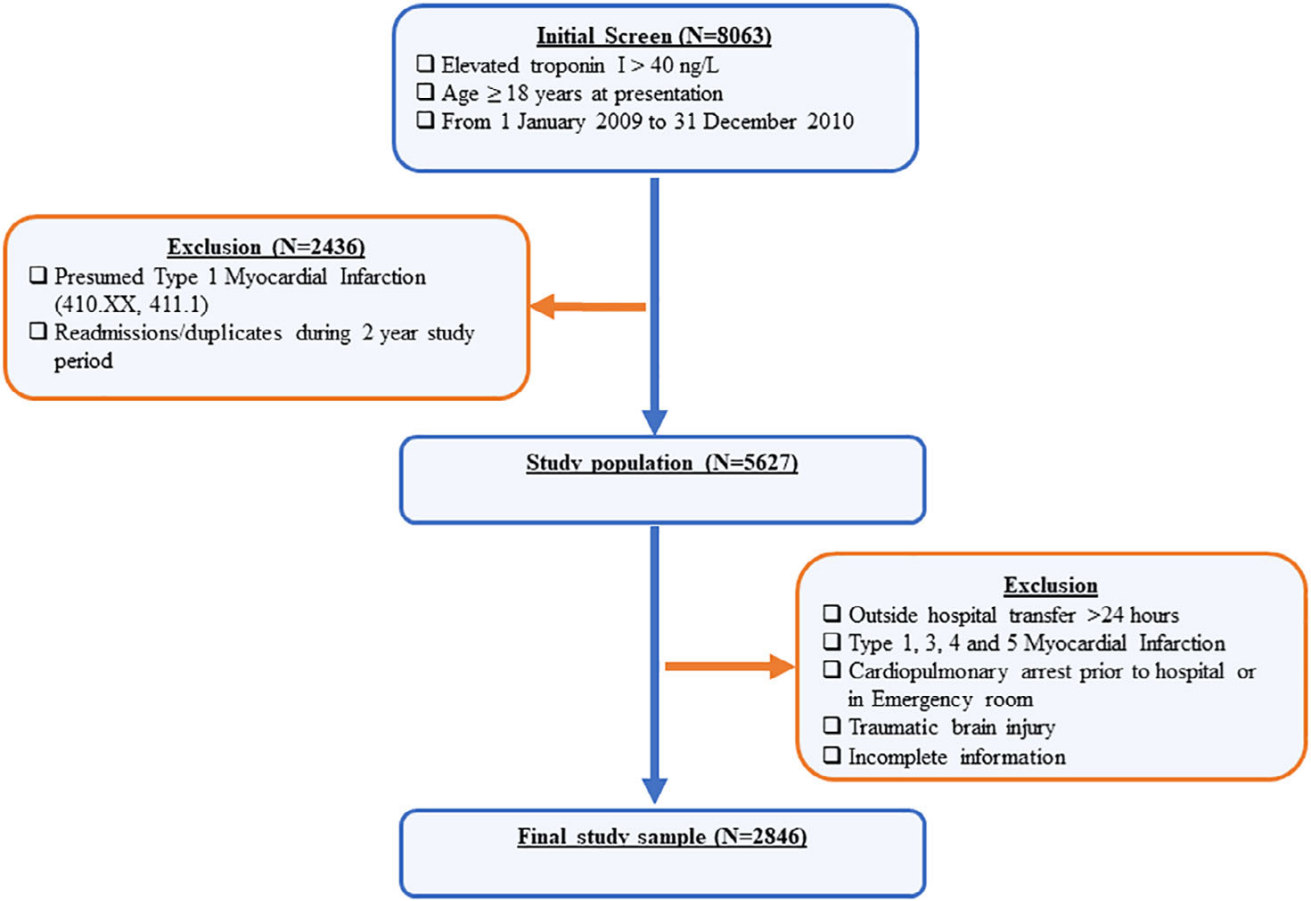
Scheme of the Wake‐Up T2MI Registry design

The Wake‐Up T2MI Registry is a registry of adults (age ≥ 18 years) who were hospitalized at Wake Forest University Medical Center in a 2‐year period between 1 January 2009 and 31 December 2010, and had T2MI or myocardial injury as defined by the Fourth Universal Definition of MI.^2^ Figure 1 provides an outline of the study design and patient selection.

### 2.1 Data source

The Clinical and Translational Science Institute (CTSI) at Wake Forest University Baptist Medical Center (WFUBMC), Winston‐Salem, North Carolina, will serve as the data source and primary organization for this registry. WFUBMC is an academic medical center with 885 licensed beds and is designated as a level I trauma center, serving 24 counties in northwest North Carolina and southwest Virginia. Electronic health records from CTSI will be accessed to study demographics, admission diagnosis, discharge diagnosis, laboratory tests, medications, medical history, procedures, and clinical notes for individuals meeting the specified criteria. Mortality data have been obtained from the National Death Index (NDI) and North Carolina State Vital Statistics from 1 January 2009 to 31 December 2017, and will be utilized for ascertainment of death as an outcome. The study protocol has been approved by the regional Internal Review Board at WFUBMC. This registry is registered in Registry of Patient Registries (RoPR) with RoPR ID number of 43530.

### 2.2 Biomarker

All troponin samples were analyzed on the Beckman DXI800 (Beckman Instruments, Beckman Coulter, Fullerton, California). This assay has a 99th percentile URL value of 30 ng/L with a coefficient of variation of 10% at 40 ng/L. We used a threshold of 40 ng/L for detection of myocardial injury during the study period.^2^, ^31^

### 2.3 Identification of study population and patient selection

Patients with elevated cTnI >40 ng/L from 1 January 2009 to 31 December 2010 were identified using data extracted from CTSI. Due to the absence of International Classification of Disease, Ninth Revision (ICD‐9) code for T2MI and myocardial injury and since administrative coding for T2MI in ICD‐10 were only available in October 2017, ascertainment of T2MI will rely on primary chart review. To limit patients with presumed T1MI, those with ICD‐9 diagnosis of acute MI (410.xx, 411.1) have been excluded from the initial inclusion criteria. The study will exclude patients with pre‐hospital cardiac arrest, patients who were transferred from an outside hospital more than 24 hours after the presentation (to limit selection of patients requiring higher levels of care to minimize the transfer of incomplete or inaccurate information), T1MI diagnosed by discharging physician, traumatic brain injury, readmission, one troponin I level, and significant missing data to sufficiently adjudicate T2MI vs myocardial injury. Data of patients who met the inclusion criteria will be entered into a secure, customized electronic adjudication system for review.

### 2.4 Ascertainment of clinical data

#### 2.4.1 Presentation

**Figure 2:**
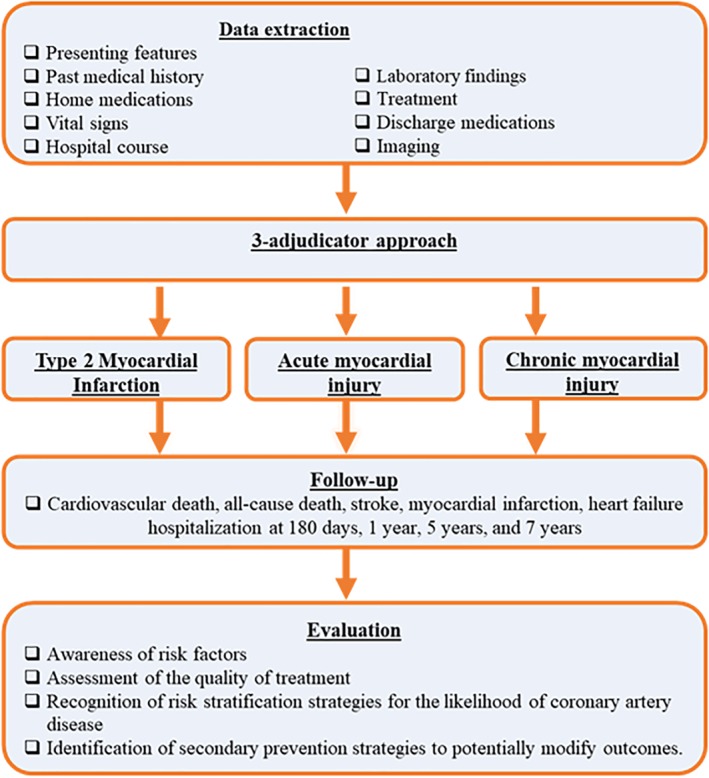
Ascertainment of clinical data in the Wake‐Up T2MI Registry

Emergency department notes or admitting physician's notes will be the source of information on the initial presentation of patients (Figure 2). Further information on variables collected during the hospital course, traditional comorbidities and risk factors, baseline medications, laboratory testing variables are provided in Appendix S1.

#### 2.4.2 Follow‐up

A follow‐up at 180 days, 1 year, 5, and 7 years of all subjects included in the registry will be conducted via a review of the electronic medical records system. At each follow‐up time‐point, health status and any interim cardiovascular (CV) events will be recorded. Vital status will be assessed via the NDI and North Carolina State Vital Statistics including the cause of death.

### 2.5 Adjudication of T2MI and myocardial injury

**Figure 3:**
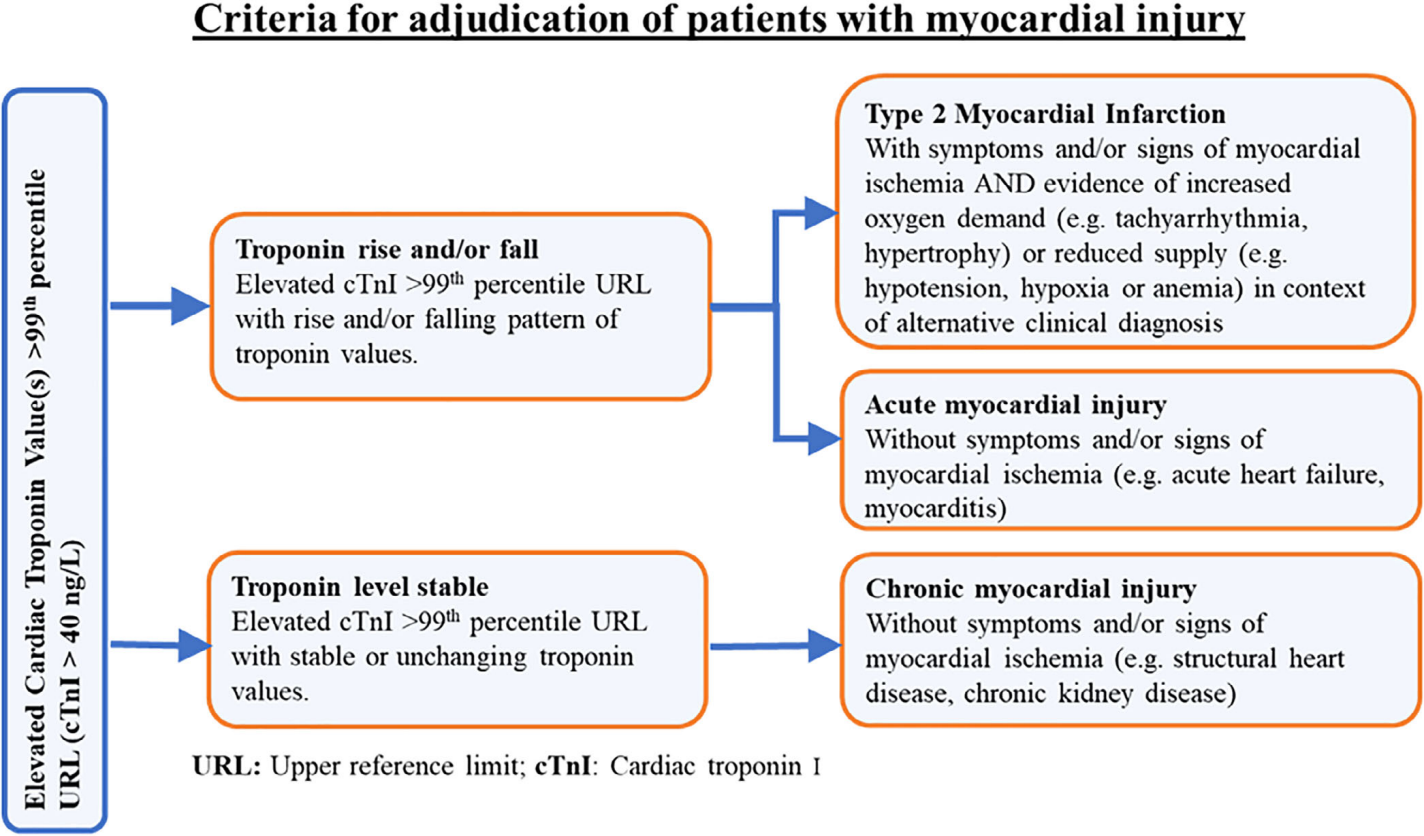
Adjudication criteria in the Wake‐Up T2MI Registry

A team of trained study physicians will review all records and utilize the Fourth Universal Definition to classify patients into (a) T2MI, (b) acute myocardial injury, and (c) chronic myocardial injury^2^ (Figure 3). All diagnoses will be adjudicated by two independent adjudicators with disagreements settled by a third adjudicator. All reviewers will have access to all available electronic patient medical records from the index admission described above. To fulfill the biomarker criteria of T2MI, an elevated cTnI of at least >40 ng/L along with an evidence of rise and/or fall of cTnI will be required. If the biomarker criteria is met, clinical conditions with potential to trigger overt ischemia along with any one of the following will be required to be diagnosed as T2MI: (a) symptoms or signs of ischemia recorded in the medical chart; (b) new or presumed new (if unknown baseline) significant ST‐segment/T‐wave changes or new left bundle branch block; (c) development of pathologic Q waves on electrocardiogram; and (d) imaging evidence of new loss of viable myocardium or new regional wall‐motion abnormality.^1^, ^2^ For a diagnosis of acute myocardial injury, an elevated cTnI of at least >40 ng/L along with an evidence of newly detected dynamic rising and/or falling pattern of cTnI and clinical conditions without overt myocardial ischemia will be required. For a diagnosis of chronic myocardial injury, an elevated cTnI of at least >40 ng/L along with stable and unchanging pattern of cTnI and clinical conditions without myocardial ischemia^2^ will be required.

### 2.6 Study endpoints

Causes of death will be classified in 1 of 3 categories (1) CV death (secondary to MI, heart failure, sudden cardiac death, stroke, CV procedure, and other CV causes such as pulmonary embolism or peripheral artery disease), (2) non‐CV death, and (3) undetermined cause of death (Appendix S2). Name, social security numbers, and date of birth will be used to match patients with the NDI/North Carolina State Vital Statistics to identified deaths at follow‐up. The definition of CV death will be adapted from the 2014 American College of Cardiology/American Heart Association key data elements and definitions for CV endpoints in clinical trials.^32^ No formal sub‐studies are planned in this study.

### 2.7 Data management

All study‐related patient data will be stored on REDCap (Research Electronic Data Capture) tools hosted by Wake Forest CTSI. REDCap is a secure, encrypted, web application for building and managing the online database. REDCap is a Health Insurance Portability and Accountability Act compliant. This web serves as an intuitive interface to enter data with real‐time validation (automated data type and range checks). This platform offers easy data manipulation with audit trails and reports for monitoring and querying of participant records.^33^

### 2.8 Statistical analysis

Continuous variables will be reported as means or medians and compared with *t* tests, Wilcoxon rank‐sum, or analysis of variance, as appropriate. Categorical variables will be reported as proportions and frequencies and will be compared with chi‐square or Fisher exact tests. Ordinal variables will be compared with a trend test. Cox proportional hazards modeling will be performed for time‐to‐event analyses. All analysis will be performed on de‐identified data. All analysis was performed using SAS software, version 9.4 (SAS Institute, Inc., Cary, North Carolina).

## 3 RESULTS

### 3.1 Baseline clinical profile of T2MI or myocardial injury

We plan to collect data from a total of 2846 patients, who met our inclusion criteria over a period of 1 January 2009 to 31 December 2010. As of November 2018, we have collected data for a total of 2205 patients. The baseline characteristics of this initial cohort are detailed in Table 3. The mean age was 65.6 ± 16.9 years, 52.2% were women, and 64% were white. Over two‐thirds (71%) had hypertension, 35% had hyperlipidemia, 30% had diabetes mellitus, and 18.5% had chronic kidney disease at baseline (Table 3). At the time of hospital admission, 40% were on aspirin, 38% were on β‐blockers, 30% were on statins, 29% were on angiotensin‐converting enzyme inhibitors, and 9% were on angiotensin II receptor blockers (Table 3).

**Table 3:** Baseline characteristics of patients in the Wake‐Up T2MI Registry (preliminary data)

Baseline characteristics	Total N = 2205
Age (years), mean (SD)	65.6 (16.9)
Male (%)	1054 (47.8)
Race	
White (%)	1409 (64)
Black (%)	753 (34)
Others (%)	43 (2)

Past medical history	
Hypertension (%)	1576 (71.5)
Diabetes (%)	659 (29.9)
Heart failure (%)	381 (17.3)
Coronary artery disease (%)	313 (14.2)
Previous myocardial infarction (%)	183 (8.3)
Prior percutaneous coronary intervention (%)	27 (1.2)
Prior coronary artery bypass graft surgery (%)	114 (5.2)
Peripheral artery disease (%)	121 (5.5)
Stroke (%)	276 (12.5)
Chronic obstructive pulmonary disease (%)	385 (17.5)
Chronic kidney disease (%)	408 (18.5)
Hyperlipidemia (%)	763 (34.6)
Smoking history (%)	1029 (46.7)
Family history of coronary artery disease (%)	584 (26.5)

Baseline medication use (N = 2205)	N (%)
Aspirin (%)	876 (39.7)
P2Y12 inhibitors (%)	110 (5)
Dual antiplatelet therapy (%)	71 (3.2)
Aspirin or P2Y12 inhibitors (%)	915 (41.5)
Beta‐blocker (%)	838 (38)
Angiotensin‐converting enzyme inhibitor (%)	639 (29)
Angiotensin II receptor blocker (%)	204 (9.3)
Statin (%)	671 (30.4)

*Note*: Continuous variables measured as mean (SD); categorical variables measured by frequency (%).

## 4 DISCUSSION

The increasing sensitivity of troponin assays, their wide ranging use, and heightened recognition by clinicians have contributed to an increase in diagnoses of T2MI and myocardial injury. These clinical entities have drawn more attention largely related to challenges in their management and poor short‐ and long‐term outcomes. To date, there have been few randomized clinical trials available to determine the effects of investigational strategies in these cohorts.^34^, ^35^ As such, observational studies defining the epidemiology of these disease entities are of great importance. In the CASABLANCA study^26^ (Catheter Sampled Blood Archive in Cardiovascular Diseases) (ClinicalTrials.gov NCT00842868), a prospective single center cohort examined 1251 patients undergoing coronary and peripheral angiographic procedures, 73.8% had at least one incident of T2MI in median of 40 months follow‐up and found 61% of T2MI had ≥50% coronary obstruction in ≥2 vessels. The role of CAD and mechanism of myocardial injury is being studied in the ongoing, prospective Determining the Mechanism of Myocardial Injury and Role of Coronary Disease in Type 2 Myocardial Infarction (DEMAND‐MI) study (ClinicalTrials.gov NCT03338504). The perioperative period is a unique clinical scenario where patients have high risk of T2MI and myocardial injury. Incidence and Outcome of Perioperative Myocardial Injury After Non‐cardiac Surgery (BASAL‐PMI) study (NCT02573532) is an ongoing observational perioperative study classifying patients into T1MI, T2MI, or myocardial injury using high‐sensitivity cTn assays. Diagnostic strategies to better characterize these entities are urgently needed, as well as targeted therapies to improve outcomes in these patients.

Upon completion of data extraction, the Wake‐Up T2MI Registry will be useful in evaluating use of cardioprotective therapies and their association with long‐term outcomes in this cohort. We would define underlying comorbid disease burden including CAD, peripheral vascular diseases, chronic obstructive pulmonary diseases, obstructive sleep apnea, obesity, hyperlipidemia, and diabetes mellitus. In addition, we would identify important risk predictors of subsequent clinical outcomes, which may help to guide development of strategies to improve outcomes in various subgroups. Furthermore, we plan to evaluate electrocardiographic, imaging, and biomarker signatures of these disease states.

The Wake‐Up T2MI Registry will generate high‐quality clinical data and its longitudinal design will enable follow‐up of short‐ and long‐term outcomes. Results from this registry may be used to structure future risk‐prediction models aimed at T2MI and myocardial injury. Ultimately, results from our registry will provide data on how to delineate better these entities, determine their cardiovascular prognosis, and potentially develop strategies to mitigate their risk. Presently, we have collected data for 2205 of the 2846 planned subjects; careful adjudication will continue to be undertaken to differentiate T2MI, acute myocardial injury, and chronic myocardial injury. Once study participants are adjudicated into gold standard diagnoses, we might better address questions regarding how baseline characteristics as a function of diagnosis, acute management, and prognosis differ.

### 4.1 Study limitations

This retrospective cohort study is subject to certain limitations inherent to its design. Our study is limited to single center in one US region, and as such, results may not be fully generalizable. To facilitate long‐term clinical follow‐up beyond 5 years, we enrolled patients in 2009 to 2010 period; this experience may thus not reflect contemporary treatment practices. Although we rely on retrospective data, individual chart review of all patients and non‐reliance on coded or administrative data fields will strengthen these data. During the period when the index hospitalizations was identified (2009‐2010), our center used conventional troponin assays; therefore, there is a potential to miss patients who could have been labeled as T2MI or myocardial injury based on high‐sensitivity assays. We used a cut‐off of troponin value >40 ng/L to obtain a coefficient of variation of 10% and therefore, we may have underestimated the number of patients with myocardial injury. Due to exclusion of all patients coded as presumed T1MI, we may miss patients who were initially clinically misidentified as T1MI (who may have T2MI or myocardial injury).

### 4.2 Conclusions

The Wake‐Up T2MI Registry will collect a large cohort of patients with T2MI and myocardial injury. By linking robust electronic health record system administrative coding, detailed chart review with independent adjudication, and national and state death records, we will obtain comprehensive data that will allow us to characterize differences in the presence and treatment of risk factors, as well as short‐ and long‐term outcomes. More granular data regarding T2MI and myocardial injury are needed to guide treatment strategies in these at‐risk populations.

## CONFLICT OF INTEREST

Authors H.R.J., S.A., A.P., P.A.C., T.H.P., M.I.A., A.D., P.R.S., W.Q., S.V., and B.U. declare no potential conflict of interests. M.V. is supported by the KL2/Catalyst Medical Research Investigator Training award from Harvard Catalyst (NIH/NCATS Award UL 1TR002541), serves on advisory boards for Amgen, AstraZeneca, Bayer AG, and Baxter Healthcare, and participates on clinical endpoint committees for studies sponsored by Novartis and the NIH. A.Q. is supported by the NHLBI T32 postdoctoral training grant (T32HL007604) and the American Heart Association Strategically Focused Research Network in Vascular Disease grant (18SFRN3390085). D.L.B. discloses the following relationships—Advisory Board: Cardax, Elsevier Practice Update Cardiology, Medscape Cardiology, PhaseBio, Regado Biosciences; Board of Directors: Boston VA Research Institute, Society of Cardiovascular Patient Care, TobeSoft; Chair: American Heart Association Quality Oversight Committee; Data Monitoring Committees: Baim Institute for Clinical Research (formerly Harvard Clinical Research Institute, for the PORTICO trial, funded by St. Jude Medical, now Abbott), Cleveland Clinic (including for the ExCEED trial, funded by Edwards), Duke Clinical Research Institute, Mayo Clinic, Mount Sinai School of Medicine (for the ENVISAGE trial, funded by Daiichi Sankyo), Population Health Research Institute; Honoraria: American College of Cardiology (Senior Associate Editor, Clinical Trials and News, ACC.org; Vice‐Chair, ACC Accreditation Committee), Baim Institute for Clinical Research (formerly Harvard Clinical Research Institute; RE‐DUAL PCI clinical trial steering committee funded by Boehringer Ingelheim), Belvoir Publications (Editor‐in‐Chief, Harvard Heart Letter), Duke Clinical Research Institute (clinical trial steering committees), HMP Global (Editor‐in‐Chief, Journal of Invasive Cardiology), Journal of the American College of Cardiology (Guest Editor; Associate Editor), Population Health Research Institute (for the COMPASS operations committee, publications committee, steering committee, and USA national co‐leader, funded by Bayer), Slack Publications (Chief Medical Editor, Cardiology Today's Intervention), Society of Cardiovascular Patient Care (Secretary/Treasurer), WebMD (CME steering committees); Other: Clinical Cardiology (Deputy Editor), NCDR‐ACTION Registry Steering Committee (Chair), VA CART Research and Publications Committee (Chair); Research Funding: Abbott, Amarin, Amgen, AstraZeneca, Bayer, Boehringer Ingelheim, Bristol‐Myers Squibb, Chiesi, Eisai, Ethicon, Forest Laboratories, Idorsia, Ironwood, Ischemix, Lilly, Medtronic, PhaseBio, Pfizer, Regeneron, Roche, Sanofi Aventis, Synaptic, The Medicines Company; Royalties: Elsevier (Editor, Cardiovascular Intervention: A Companion to Braunwald's Heart Disease); Site Co‐Investigator: Biotronik, Boston Scientific, St. Jude Medical (now Abbott), Svelte; Trustee: American College of Cardiology; Unfunded Research: FlowCo, Fractyl, Merck, Novo Nordisk, PLx Pharma, Takeda. J.L.J. is supported in part by the Hutter Family Professorship, and has received grant support from Roche Diagnostics, Abbott Diagnostics, Singulex, Prevencio, Novartis and Cleveland Heart Labs, consulting income from Roche Diagnostics, Abbott, Prevencio and Critical Diagnostics and participates in clinical endpoint committees/data safety monitoring boards for Siemens Diagnostics, Novartis, Bayer, AbbVie and Amgen. D.H. receives grant support from the NIH and serves as a consultant to Amarin Corporation.

## Supporting information


**Appendix S1**. Definitions and data collection.
**Appendix S2**. Excluded ICD‐9 codes: 410.xx, 411.1.
