# Supplementary material for: Wake Forest University long‐term follow‐up of type 2 myocardial infarction: The Wake‐Up T2MI Registry
Source: Clin Cardiol. 2019 Apr 12;42(6):592–604. doi: 10.1002/clc.23182 (PMC6553563; doi:10.1002/clc.23182)
Supplement: Supplementary file 1 — Appendix S1. Definitions and data collection. Appendix S2. Excluded ICD‐9 codes: 410.xx, 411.1. [file CLC-42-592-s001.docx]

**Wake Forest University Long-Term Follow-up of Type 2 Myocardial Infarction:**

**The Wake-Up T2MI Registry**

**Supplemental Material**

**Supplemental Appendix 1.**

**Definitions:**

**Acute Myocardial Injury**: Elevated cTnI concentration above the upper reference limit (myocardial necrosis) along with raise and/or fall pattern, without any symptoms OR signs of myocardial ischemia in the context of alternative clinical diagnosis. Example: Acute heart failure, myocarditis

**Chronic Myocardial Injury**: Elevated cTnI concentration above the upper reference limit with stable or unchanging troponin pattern, without any symptoms OR signs of myocardial ischemia. Example: Structural heart disease, chronic kidney disease

**Type 2 Myocardial Infarction**: Elevated cTnI concentration above the upper reference limit (myocardial necrosis) along with raise and/or fall pattern, with symptoms and/or signs of myocardial ischemia AND evidence of increased oxygen demand (e.g. tachyarrhythmia, left ventricular hypertrophy) or reduced supply (e.g. hypotension, hypoxia, anemia) in the context of alternative diagnosis.

**Data Collection:**

**Hospital Course**: Each patient’s hospital course will be reviewed, including progress notes, acute events, consult notes, laboratory findings, and findings on both noninvasive and invasive imaging. We will ascertain treatments, cardiovascular (CV) medications, and identify all in-hospital complications, including but not limited to new-onset arrhythmias, new or exacerbation of heart failure, surgical complications, and in-hospital death during the index hospitalization.

**Outside facility transfers**: Details on whether patient arrived directly or transferred from another facility will be collected. For the patients who were transferred from an outside hospital less than 24 hours after admission to outside hospital, symptoms and vital signs at presentation to the outside hospital will be recorded from the transfer notes contingent upon availability.

**Comorbidities and Risk Factors**: Medical records will be reviewed to extract traditional CV risk factors and key comorbidities. History of smoking (current or former), alcohol (current or former), and recreational drug use (current or former), as documented by admission notes or detected on toxicology will be extracted as well. Family history of CAD will be defined as any first-degree relative with a history of fatal/nonfatal MI or having undergone coronary revascularization.

**Baseline Medications:** We will extract data regarding the type and doses of CV medications (antiplatelets, anticoagulants, statins, beta-blockers, renin-angiotensin-aldosterone-system inhibitors, diuretics, nitrates, phosphodiesterase 5 inhibitors and calcium channel blockers) at presentation and discharge. We will also ascertain information on medications administered during hospitalization such as antibiotics, new anticoagulants, intravenous diuretics, and vasopressors/inotropes.

**Laboratory Testing:** Laboratory values during admission will be reviewed including peak and lowest cTnI level, white blood cell count, admission hemoglobin and lowest hemoglobin, estimated glomerular filtration rate, glycated hemoglobin, cholesterol parameters, natriuretic peptides, urine drug screen, and blood and urine cultures during hospitalization.

**Cardiovascular Diagnostic Evaluation**: Electrocardiogram parameters on admission or during any acute event as read by cardiologist will be reviewed including, rhythm, son-specific ST or T wave changes, T wave inversion, hyperacute T waves, ST depression, ST elevation, and atrial fibrillation/atrial flutter. ECHO cardiogram variable will be reviewed including, ejection fraction, valve abnormalities, pulmonary artery pressure, left ventricular hypertrophy, diastolic abnormalities, and regional wall motion abnormalities. Reviewed any cardiac invasive procedures.

**Supplemental Appendix 2:**

Excluded ICD-9 codes: 410.xx, 411.1

**Classification of Cardiovascular Death ICD-10 Code Definition**

Ischemic heart diseases

I20 Angina pectoris

I21 Acute myocardial infarction

I22 Subsequent myocardial infarction

I23 Certain current complications from acute myocardial infarction

I24 Other acute ischemic heart diseases

I25 Chronic ischemic heart disease

Other forms of heart disease

I34 Non-rheumatic mitral valve disorders

I35 Non-rheumatic aortic valve disorders

I36 Non-rheumatic tricuspid valve disorders

I37 Pulmonary valve disorders

I42 Cardiomyopathy

I43 Cardiomyopathy in diseases classified elsewhere

I46 Cardiac arrest

I48 Atrial fibrillation and flutter

I49 Other cardiac arrhythmias

I50 Heart failure

I51 Complications and ill-defined descriptions of heart disease

Cerebrovascular diseases

I60 Subarachnoid hemorrhage

I61 Intracerebral hemorrhage

I62 Other nontraumatic intracerebral hemorrhage

I63 Cerebral infarction

I64 Stroke, not specified as hemorrhage or infarction

I65 Occlusion and stenosis of precerebral arteries, not resulting in infarction

I66 Occlusion and stenosis of cerebral arteries, not resulting in infarction

I67 Other cerebrovascular diseases

I68 Cerebrovascular disorders in diseases classified elsewhere

I69 Sequelae of cerebrovascular disease

Thank you for your consideration.

Sincerely,

Hanumantha R. Jogu, MD
